# Application of Graphene Hybrid Materials in Fault Characteristic Gas Detection of Oil-Immersed Equipment

**DOI:** 10.3389/fchem.2018.00399

**Published:** 2018-09-11

**Authors:** Lingfeng Jin, Weigen Chen, Ying Zhang

**Affiliations:** ^1^State Key Laboratory of Power Transmission Equipment & System Security and New Technology, Chongqing University, Chongqing, China; ^2^School of Electrical Engineering, Chongqing University, Chongqing, China; ^3^School of Electrical and Computer Engineering, Georgia Institute of Technology, Atlanta, GA, United States

**Keywords:** graphene, gas sensor, oil-immersed equipment, sensing mechanism, fault characteristic gas

## Abstract

Graphene and its hybrid materials, due to their unique structures and properties, have attracted enormous attention for both fundamental and applied research in the gas sensing field. This review highlights the recent advances in the application of graphene-based gas sensors in fault characteristic gas detection of oil-immersed equipment, which can effectively achieve condition monitoring of the oil-immersed power equipment. In this review, the synthetic methods of graphene hybrid materials with noble metals, metal oxides and their combination are presented. Then, the basic sensing mechanisms of graphene hybrid materials and gas sensing properties of graphene hybrid materials sensors to hydrogen (H_2_), carbon monoxide (CO), carbon dioxide (CO_2_), methane (CH_4_), acetylene (C_2_H_2_), ethylene (C_2_H_4_), and ethane (C_2_H_6_), which are the fault characteristic gas in oil-immersed power equipment, are summarized. Finally, the future challenges and prospects of graphene hybrid materials gas sensors in this field are discussed.

## Introduction

Graphene is an allotrope of carbon consisting of a single layer of carbon atoms arranged in a hexagonal lattice, which was rediscovered, isolated, and characterized by Geim and Novoselov in 2004 (Novoselov et al., 2004; Geim and Novoselov, 2007). Owing to the unique structure, graphene exhibits excellent physical and chemical properties, and has opened a new and very promising scientific area with a lot of focus on material science and potential applications (Aïssa et al., 2015; Higgins et al., 2016; Long et al., 2018). Among these outstanding properties of graphene, the high electron mobility of up to 200,000 cm^2^/Vs and superior specific surface area of 2,630 m^2^/g make graphene an extremely sensitive material for gas detection (Akturk and Goldsman, 2008; Chen et al., 2008; Bonaccorso et al., 2015). However, pristine graphene, which lacks dangling bonds, is unfavorable for the adsorption of gas molecules on its surface. Therefore, the modification of graphene and its derivatives via physicochemical methods, such as covalent, non-covalent and doping functionalization, has been investigated. Usually, graphene hybrid materials, which are composed of graphene and typical sensing materials (e.g., noble metals, metal oxides, or their ternary hybrids), have significantly improved performance due to the synergistic interaction between graphene and the typical sensing material (Meng et al., 2015). There have been numerous works published on the basic research and the sensing applications of graphene and graphene hybrid materials.

One such application is condition monitoring of oil-immersed equipment. Insulating oil with high specific heat and dielectric strength is widely used in the high voltage power equipment. However, the insulating oil can be cracked into small related gas molecules due to the long-term electrothermal effect, which reduces the insulation strength and leads to the malfunction of the power equipment. According to previous studies, effective detection of seven typical fault characteristic gases, including H_2_, CO, CO_2_, CH_4_, C_2_H_2_, C_2_H_4_, and C_2_H_6_, can reflect the operation state of oil immersed power equipment (Bakar et al., 2014). Recently, a lot of research has been carried out on using graphene hybrid materials to detect these gases for rapid and accurate fault detection of oil-immersed equipment (Acharyya and Bhattacharyya, 2016; Zhou et al., 2016; Nasresfahani et al., 2017; Zhang et al., 2017b).

Although there are many reviews on the applications of gas sensing using graphene materials (Wang T. et al., 2016; Singhal et al., 2017; Dai et al., 2018), few has focused on detecting the typical gases reflecting the characteristics of faults in oil-immersed equipment. The objective of this review is to provide researchers a systematic understanding of the development of graphene hybrid materials in this application field. The main synthesis method and properties of graphene hybrid materials are introduced in section Synthesis, Properties, and Experimental Testing. Then, the gas sensing mechanism of graphene hybrid materials is discussed in section Sensing Mechanism. In section Application of Gas Sensor, the gas sensing properties of graphene hybrid materials to typical fault characteristic gases of oil-immersed equipment are summarized and compared. Finally, we analyze the challenges of gas sensing application in this field and present the suggestions of the future development trend.

## Synthesis, properties, and experimental testing

### Synthesis and properties

Since the discovery of graphene in 2004, a lot of research has been conducted on how to synthesize graphene with high quality and large scale. To this date, the preparation of graphene can be mainly divided into top-down routes and bottom-up routes. Top-down routes use graphite as raw materials, and graphene is mainly obtained through separating the carbon atom layer by mechanical exfoliation, chemical oxidation-reduction reaction, and electrochemical methods. In contrast, bottom-up routes use small carbon-based molecules as raw materials, and graphene is obtained by silicon carbide pyrolysis, chemical vapor deposition (CVD), and solvothermal methods. Paton et al. (2014) developed a simple model that shows exfoliation occurs once the local shear rate exceeds 10^4^/s and demonstrated a scalable method for producing relatively large quantities of defect-free graphene. Rathnayake et al. (Yola et al., 2015) presented the methods for preparing graphene oxide (GO) and reduced graphene oxides (rGO) using chemical oxidation and reduction processes, respectively, which are based on needle platy variety of natural vein graphite that has high purity and crystallinity and low cost. Hofmann et al. (2015) investigated the process of electrochemical exfoliation and the impact of its parameters on the produced graphene, and achieved the synthesis of graphene with controllable electronic and mechanical characteristics. Son et al. (2015) reported direct graphene growth over silicon nanoparticles without silicon carbide formation, and the volumetric energy densities are 972 and 700 Wh/L at the first and 200th cycles, respectively. Banszerus et al. (2015) showed that the quality of CVD-grown graphene depends critically on the used transfer process and reported an advanced transfer technique that allows both reusing the copper substrate of the CVD growth and making devices with mobilities as high as 350,000 cm^2^/Vs. Quan et al. (2014) successfully synthesized sulfur-doped and nitrogen-doped graphene by using a solvothermal method, and these heteroatom-doped graphene materials exhibited high surface areas and high contents of heteroatoms. With the synthetic routes mentioned above, controllable preparation of graphene can be achieved.

Graphene as an ideal base material can be combined with noble metals, metal oxides, or their ternary hybrids to form graphene hybrid materials with outstanding gas sensing properties. Metal nanoparticles combine the excellent properties of metal and characteristics of nanomaterials, and show great potential in catalysis, sensing and electronics fields. Up to now, nanoparticles of many different metals, such as Au, Ag, Pd, Pt, Cu, Ni, Co, have been successfully combined with graphene. Phan et al. (in press) synthesized Pt nanoparticle-loaded 3D graphene for H_2_ sensing using a polymer-assisted hydrothermal method and the H_2_ sensor had a response value of 16% and response/recovery times of 9/10 s with a 1% H_2_ concentration at 200 °C. Semiconductor metal oxide such as SnO_2_ (Wang et al., 2015), ZnO (Zhu and Zeng, 2017), TiO_2_ (Zhang et al., 2018), etc. are used widely in gas detection. The hybrids of graphene and metal oxides are mainly prepared by *in-situ* synthesis, liquid phase method and hydrothermal method. Ye et al. (2015) developed porous graphene embedded with various types of metal oxide nanoparticles through direct laser scribing on the metal-complex-containing polyimide film. Zhang B. et al. (2017) prepared rGO/α-Fe_2_O_3_ hybrids with different rGO contents, which were composed of the round-edged cubic α-Fe_2_O_3_ particles adhering uniformly on both sides of the crumpled and rippled rGO sheets. The local p-n heterojunctions between n-type α-Fe_2_O_3_ and p-type rGO caused an extension of electron depletion layer and potential barriers, which in turn led to significant resistance variation. Zhou et al. (2017) synthesized rGO and rGO/ZnO thin films and the performances of sensing organic vapor molecules are enhanced. In order to combe the advantages of noble metal and metal oxide, the ternary graphene hybrid materials have been developed. Uddin et al. (2015a) synthesized an Ag/ZnO/rGO hybrid via photochemical method and the 5 wt% hybrid enhanced the C_2_H_2_ sensing performance. Wei et al. (2017) synthesized the composite of Ag/SnO_2_/rGO via a hydrothermal reaction process with a high surface area of 191.583 m^2^/g and showed enhanced sensing properties to ethanol. Esfandiar et al. (2014) synthesized Pd/WO_3_/ rGO as hybrid sensing material via the facile hydrothermal method and an improvement in H_2_ sensing at low temperatures was observed.

### Sensor fabrication and testing

**Figure 1:**
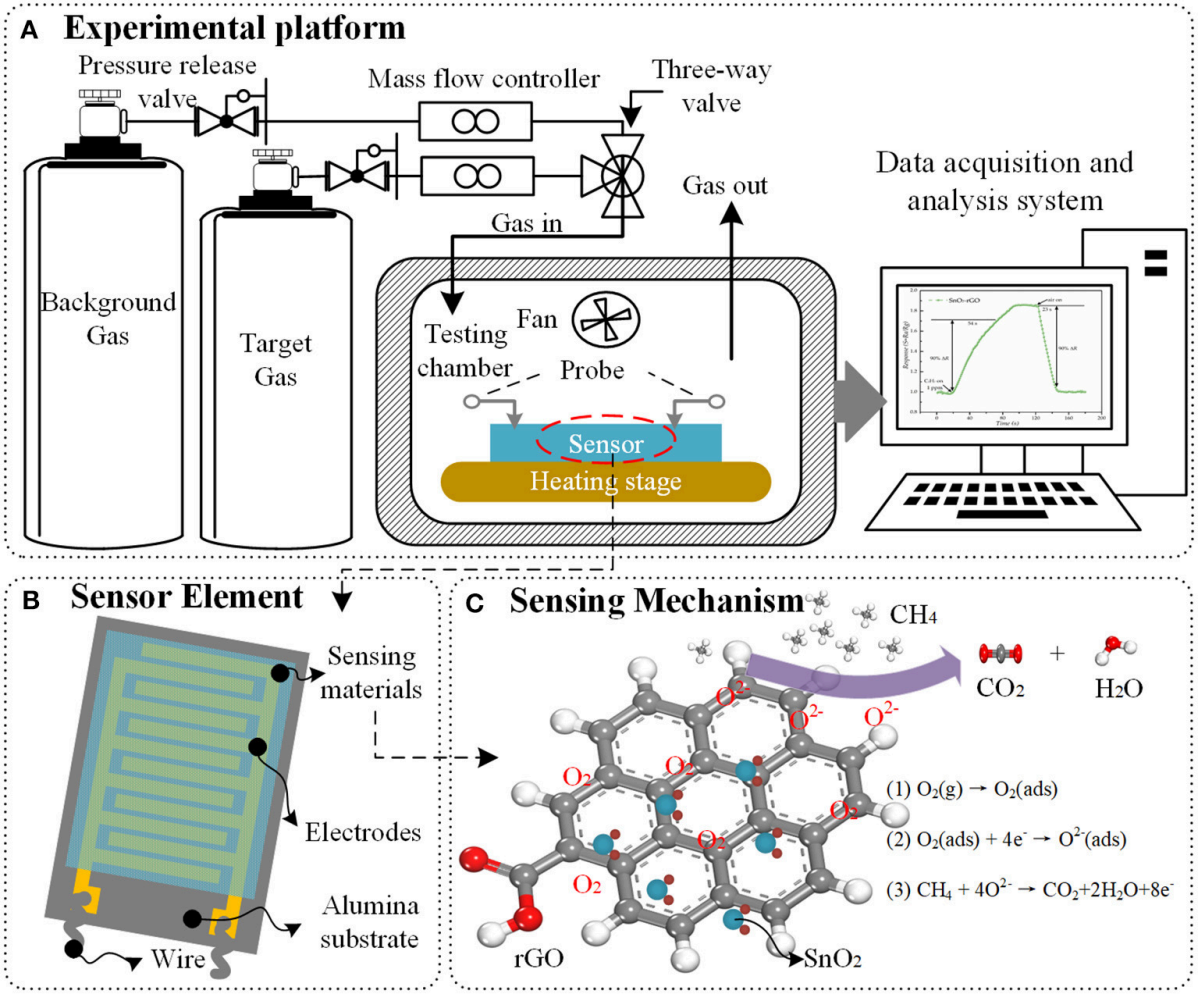
Schematic illustration of **(A)** gas sensing experimental platform, **(B)** structure of planar sensor element, and **(C)** sensing mechanism between SnO_2_/rGO hybrid materials and methane.

Graphene hybrid materials can be used to fabricate gas sensors. Based on the operating mode, the device structure of gas sensor can be classified into directly heated and indirectly heated types. For a directly heated sensor, the sensing materials are in direct contact with the heater, which may make the sensor lose its stability and anti-interference ability. Therefore, the indirectly heated sensors are most widely used in scientific research and commerce, as shown in Figure 1B. The planar gas sensor is usually composed of three layers: sensing materials, detection electrodes and substrate (Nguyen et al., 2014). The synthesized sensing materials are covered by the detection electrodes. The interdigital electrodes are used to measure the resistance of the sensing materials. The substrate, made of silicon or alumina, has a good compatibility with integrated circuits and can support the sensing materials.

The responses of the sensor to different gases are carried out by using the gas sensing experimental platform, which composed of gas sources, MFCs (mass flow controllers), testing chamber and computer-controlled data acquisition and analysis system, as presented in Figure 1A. The experimental procedure is as follows: firstly, the sensor is placed in the center of the heating stage with two adjustable probes. Then, the heater starts to work and the background gas is delivered into the sealed chamber with a constant speed. When the resistance of the sensor no longer changes, the test chamber is filled with a different concentration of the gas being tested. At last, the background gas will be delivered again after the response of the sensor becomes stable. The response of sensor can be defined as a function of the change of resistance value of sensor (Barsan and Weimar, 2003). All the experiments need to be carried out under the same ambient temperature and relative humidity.

## Sensing mechanism

Schedin et al. reported that the graphene gas sensor can effectively detect the adsorption or dissociation behavior of a single gas molecule on the surface of graphene, due to the fact that the change of carrier concentration of graphene leads to the change in electrical conductivity (Schedin et al., 2007). Meanwhile, due to the existence of oxygen functional groups, GO and rGO usually show better sensing properties than graphene. When GO or rGO are exposed to the air, the oxygen functional groups of them mainly combine H_2_O molecules with hydrogen bonds and the adsorbed H_2_O molecules are transferred to H_3_O^+^, further promoting the formation of ion channels on the surface of the sample. When they are in contact with adsorbed molecules, the hydrogen bonds will be destroyed, which inhibits the ionization reaction between oxygen functional groups and H_2_O molecules and leads to step-like changes in resistance (Ozcan et al., 2018). Prezioso et al. (2013) prepared a p-type gas sensor through GO drop-cast on standard interdigitated Pt electrodes and its sensing properties to NO_2_ was analyzed. They also presented the sensing mechanism of the gas sensor: when NO_2_ molecules adsorbed on the oxygen functional groups, the electrons of the adsorption sites are transferred to NO_2_ molecules, which leads to the decrease of electron concentration in the surface of the sensing materials, giving a reason for the p-type behavior.

In general, the sensing mechanisms of graphene hybrid are analyzed from the following three aspects. First, the introduction of nanoparticles can effectively prevent the aggregation of graphene sheets, thereby the graphene hybrid material is more favorable to form a 3D porous nanostructure with higher specific surface area, and more adsorption sites, vacancies, defects, and sp^2^-bonded carbon, which are beneficial to the adsorption of gas molecules (Russo et al., 2012; Zhang et al., 2015a). Secondly, the formation of p-n heterojunctions between graphene and metal/metal oxide enhances the gas sensing properties. Once the target gas molecules are in contact with these interfaces, the depletion layers at the heterojunctions will be modulated, the electron state will be changed, and the phenomenon of charge transfer is more active, which lead to a larger relative change of resistance of graphene hybrid material (Tran et al., 2014). Thirdly, when hybrid materials contain metal oxide (e.g., SnO_2_, ZnO, CuO, Co_3_O_4_), the sensing behavior can be explained by the surface-adsorbed oxygen (Bai et al., 2015). For n-type metal oxide material, the oxygen molecules O_2(gas)_ will capture electrons from the surface of metal oxide to form chemisorbed oxygen species (${\text{O}}_{2}^{-}$, O^−^, or O^2−^), which leads to a high resistance of the sensor, as observed in the experiments (Jin et al., 2016). As shown in Figure 1C, when the sensor is exposed to reducing gas such as methane, the target gas molecules will react with chemisorbed oxygen species and obtain electrons from them, which reduces the concentration of electron on the surface of the sensing materials. Obviously, the gas sensing reaction is a ticklish issue associated with the intricate nanostructure and complicated sensing mechanism (Wang et al., 2011; Sun et al., 2015; Wang Z. et al., 2016). The research on the characterization methods and simulation analysis from atomic level provide a new perspective and starting point for the study on the sensing mechanism of nanomaterials.

## Application of gas sensor

Graphene hybrid materials exhibit excellent sensing properties to H_2_, CO, CO_2_, and small hydrocarbon gas (e.g., CH_4_, C_2_H_2_, C_2_H_4_, C_2_H_6_). In this section, we summarize and discuss the related works based on the recently published papers (Table 1).

**Table 1:** Summary of recent researches on graphene hybrid materials sensor for sensing of fault characteristic gases in oil-immersed equipment.

**Gas**	**Hybrid material**	**Temp. (°C)**	**Detection range (μL/L)**	**Conc. (μL/L)**	**Response type**	**Sensor response**	**τ_res_/τ_rec_ (s/s)**	**References**
H_2_	Pd/G	RT	1,000	1,000	Δ*G*/*G*_air_	26%	40/490	Alfano et al., 2017
	Pt/G	320	1,000–20,000	10,000	Δ*R*/*R*_air_	1.6%	~1/0.72	Harley-Trochimczyk et al., 2015
	Pd/Ag/G	105	100-5,000	500	Δ*R*/*R*_air_	9.96%	102/–	Sharma and Kim, 2018
	MoO_3_/G	RT	0.5–1,000	1,000	*R*_air_/*R*_gas_	20.5	~10/30	Yang et al., 2017
	CuO/rGO/CuO	RT	50–1,500	100	Δ*R*/*R*_air_	4.2%	<80/60	Zhang et al., 2017c
	Pd/WO_3_/G	RT	1,000–5,0000	1,000	Δ*I*	12 μA	~17/–	Chen et al., 2018
CO	rGO	RT	10–30	30	Δ*R*/*R*_air_	~71%	<30/–	Panda et al., 2016
	NiO/G	100	5–100	100	Δ*R*/*R*_air_	~120%	20/152	Khaleed et al., 2017
	CuO/rGO	RT	0.25–1,000	1	Δ*R*/*R*_air_	2.56%	70/160	Zhang et al., 2017a
	ZnO/rGO	200	1–1,000	1,000	Δ*R*/*R*_air_	85.2%	9/10	Ha et al., 2018
	GdInO_3_/rGO	90	20–100	20	Δ*R*/*R*_gas_	48%	14/15	Balamurugan et al., 2016
	Pd/SnO_2_/rGO	RT	50–1,600	1,500	Δ*R*/*R*_air_	4%	70/80	Shojaee et al., 2018
CO_2_	rGO	RT	100–1,000	1000	Δ*R*/*R*_air_	1.65%	–	Nemade and Waghuley, 2014b
	rGO	RT	0–1,500	1,500	Δ*R*/*R*_air_	71%	~4 min	Nemade and Waghuley, 2014a
	Sb_2_O_3_/G	RT	0–50	50	Δ*R*/*R*_air_	~22%	16/22	Wu et al., 2016
	Al_2_O_3_/G	125	0–200	100	Δ*R*/*R*_air_	~8.1%	14/22	Hafiz et al., 2014
	Y_2_O_3_/G	RT	0–35	35	Δ*R*/*R*_air_	1.08%	–	Nemade and Waghuley, 2013
CH_4_	PANI/rG	RT	10–3,200	100	*R*_air_/*R*_gas_	~3	85/45	Wu et al., 2013
	NiO/rGO	260	100–6,000	100	Δ*R*/*R*_air_	~2.2%	6/16	Zhang et al., 2016
	ZnO/rGO	190	100–4,000	1000	Δ*R*/*R*_air_	~12%	~200	Zhang et al., 2015b
	SnO_2_/rGO	150	1,000–10,000	1000	Δ*R*/*R*_air_	47.6%	61/330	Navazani et al., 2018
	Pd/SnO_2_/rGO	RT	800–16,000	14,000	Δ*R*/*R*_air_	9.8%	5/7 min	Nasresfahani et al., 2017
C_2_H_2_	SnO_2_/rGO	180	0.5–500	50	*R*_air_/*R*_gas_	12.4	54/23	Jin et al., 2016
	Ag/ZnO/rGO	150	1–1,000	100	*R*_air_/*R*_gas_	21.2	25/80	Uddin et al., 2015b
	Ag/SnO_2_/rGO	90	5–500	50	Δ*R*/*R*_air_	15.44	235/160	Jiang et al., 2017

*G, graphene; rGO, reduced graphene oxide; PANI, polyaniline; RT, room temperature; ΔI, which is calculated as the current change of gas sensitive response; ΔG = |G_air_ - G_gas_|, where G_gas_ is the conductance exposure to target gas concentration and G_air_ is the conductance exposure to air or nitrogen; ΔR = |R_air_ - R_gas_|, where R_gas_ is the resistance exposure to target gas concentration and R_air_ is the resistance exposure to air or nitrogen*.

### Hydrogen

The amount of H_2_ in the insulating oil will increase significantly before the electrical fault or thermal failure occurs. Therefore, online monitoring of the H_2_ content can ensure the security and stability of operation of oil immersed equipment. Noble metal is known for its high superior selectivity for the adsorption of H_2_ due to its catalytic activity for H_2_ molecules. The gas sensor based on Pd (Alfano et al., 2017) or Pt (Harley-Trochimczyk et al., 2015) nanoparticles loaded graphene demonstrated high sensitivity to H_2_ with short response and recovery time. Sharma and Kim (2018) fabricated a MEMS H_2_ sensor based on Pd-Ag/graphene, which showed a detection limit of 500 μL/L due to the phase transition of Pd-Ag. In addition, as metal oxide exhibits excellent gas sensing properties for H_2_, the graphene-metal oxide hybrid materials are usually used to detect H_2_ with low limit of detection (LOD) at room temperature. Wang et al. synthesized MoO_3_ nanoribbon/graphene hybrid for H_2_ sensing with ultralow LOD of 0.5 μL/L (Yang et al., 2017). Zhang et al. (2017c) reported a high-performance H_2_ gas sensor based on CuO/rGO/CuO sandwiched nanostructure. Chen and co-workers successfully fabricated a device based on gasochromic-Pd/WO_3_/graphene/Si tandem structure with fast response and recovery time for H_2_ (Chen et al., 2018).

### Carbon monoxide and carbon dioxide

CO and CO_2_ are mainly associated with the presence of overheating fault, which are decomposed from the cellulose of insulating oil paper. Consequently, development of high-performance CO and CO_2_ gas sensors is an effective way to monitor the insulation performance of oil-immersed equipment. The gas sensors based on rGO (Hafiz et al., 2014; Panda et al., 2016; Wu et al., 2016) can lower the operating temperature to room temperature. The graphene metal oxide hybrid materials such as NiO/graphene (Khaleed et al., 2017), CuO/rGO (Zhang et al., 2017a) ZnO (Ha et al., 2018), can detect CO with low LOD (as low as 1 μL/L), and quick response and recovery time (9 s/10 s). Balamurugan et al. (2016) demonstrated a selective CO sensor based on rGO decorated mesoporous hierarchical GaInO_3_, which exhibited a high response rate of 48% to 20 μL/L CO and appreciably fast response (~14 s) and recovery (~15 s) at 90°C. Shojaee and co-workers synthesized Pd-loaded SnO_2_/rGO hybrid materials by a hydrothermal method for CO sensing application (Shojaee et al., 2018). Additionally, much research on CO_2_ sensor based on graphene hybrid materials have been carried out by Nemade's group. They have fabricated sensors with excellent stability, low LOD and short response and recovery time within 30 s based on Sb_2_O_3_ quantum dots (QDs)/graphene (Nemade and Waghuley, 2014b), Al_2_O_3_ QDs/graphene (Nemade and Waghuley, 2014a), and Y_2_O_3_ QDs/graphene (Nemade and Waghuley, 2013).

### Hydrocarbon gas

The hydrocarbon gas (CH_4_, C_2_H_2_, C_2_H_4_, and C_2_H_6_) may be generated when electrical or thermal failures occur, such as oil or high-temperature overheating, partial discharge, spark discharge, or arc discharge. These four hydrocarbon gases have a small difference in molecular structure and chemical composition, and the sensing materials usually have the similar response to them. According to the relationship between principle gases and associated fault types (Bakar et al., 2014), when the oil-immersed equipment is suffering an electric or heating fault under any condition, the produced hydrocarbon fault characteristics gas is mainly CH_4_ or C_2_H_2_, and the amount of each of them is larger than high molecular weight gases (C_2_H_4_ and C_2_H_6_) (Sun et al., 2017). Moreover, the detection of CH_4_ or C_2_H_2_ is becoming more and more important in many fields as the mining industry, environment monitoring and petrochemical industry. Therefore, the research on the detection of hydrocarbon gas in oil-immersed equipment is mainly focused on CH_4_ and C_2_H_2_. Wu et al. reported a CH_4_ sensor based on polyaniline (PANI)/graphene. Due to the existence of the π-π^*^ conjugation system within the PANI/graphene, this sensor showed a high sensitivity of 10–3,200 μL/L at room temperature (Wu et al., 2013). Zhang et al. (2016) prepared NiO/rGO hybrid materials and demonstrated a high selectivity toward CH_4_ against H_2_, CO, and CO_2_. The response to 100 μL/L CH_4_ was 2.2% at 260°C. Graphene hybrid with ZnO (Zhang et al., 2015b) and SnO_2_ (Navazani et al., 2018) also exhibited unique sensing properties to CH_4_ around 190 and 150°C. Nasresfahani et al. (2017) synthesized Pd-doped SnO_2_/rGO via hydrothermal route, and the sensing performance to 800–16,000 μL/L CH_4_ were carried out at room temperature. Gas sensing characteristics of C_2_H_2_ sensor based on SnO_2_/rGO were carried out for 0.5–500 μL/L C_2_H_2_ at 180°C. The *p*-*n* heterojunctions between SnO_2_ and rGO, oxygen functional groups and high specific surface area of SnO_2_/rGO hybrid materials contribute to the high performance of sensor (Jin et al., 2016). Furthermore, Ag/ZnO/rGO (Uddin et al., 2015b) and Ag/SnO_2_/rGO (Jiang et al., 2017) hybrid materials were synthesized, and the sensors were developed for low-temperature C_2_H_2_ sensing. However, with the decrease of operating temperature, the sensitivity of the sensor was reduced with longer response-recovery time.

## Conclusions

Graphene hybrids are distinctive and promising sensing materials for detection of various gases, due to their ultrahigh electron mobility and large specific surface area. Compared to other types of sensor, the graphene-based sensors exhibit excellent properties and provide a new idea for high sensitivity detection of the oil-immersed power equipment at low temperature.

Although great progress has been made in this field, there are still many challenges that need to be addressed. First, lower LOD and faster response-recovery time are needed. Graphene-based sensors provide a possible approach to detect gases at room temperature, but the reaction activities between gas molecules and sensing materials may be reduced, especially during the desorption, making it difficult to meet the engineering requirements. Ultraviolet light assisted excitation technology and novel multi-dimensional hierarchical nanostructure of graphene hybrid materials are useful ways to solve this problem. Secondly, avoiding the cross-sensitivity in the detection of mixed gas. Gas sensors usually exhibit similar responses toward different gases of different concentrations. The functionalization of graphene, operating temperature modulation and design of filter layer are workable methods to overcome this challenge. Moreover, gas sensor arrays, composed of group of sensors and signal processing algorithms, can realize the qualitative identification and quantitative detection of mixed gas. Thirdly, developing more stable materials and sensors. In practical application, the condition of detection environment, such as vibration, temperature, humidity, and other contaminants, may affect the sensitivity of sensors. The improvement of sensing materials, coating method, and fabrication techniques can avoid or reduce the impact of these factors. Finally, the development in this field is inseparable from the research of new materials as well as the corresponding sensing mechanism.

## Author contributions

WC and LJ summarized and analyzed the related literature. LJ wrote the manuscript under the guidance of YZ and WC. All authors read and approved the manuscript.

### Conflict of interest statement

The authors declare that the research was conducted in the absence of any commercial or financial relationships that could be construed as a potential conflict of interest.
